# Classification of Game Demand and the Presence of Experimental Pain Using Functional Near-Infrared Spectroscopy

**DOI:** 10.3389/fnrgo.2021.695309

**Published:** 2021-12-21

**Authors:** Stephen H. Fairclough, Chelsea Dobbins, Kellyann Stamp

**Affiliations:** ^1^School of Psychology, Liverpool John Moores University, Liverpool, United Kingdom; ^2^School of Information Technology and Electrical Engineering, The University of Queensland, Brisbane, QLD, Australia; ^3^School of Computer Science and Mathematics, Liverpool John Moores University, Liverpool, United Kingdom

**Keywords:** fNIRS, games, pain, ECG, neuroadaptive technology

## Abstract

Pain tolerance can be increased by the introduction of an active distraction, such as a computer game. This effect has been found to be moderated by game demand, i.e., increased game demand = higher pain tolerance. A study was performed to classify the level of game demand and the presence of pain using implicit measures from functional Near-InfraRed Spectroscopy (fNIRS) and heart rate features from an electrocardiogram (ECG). Twenty participants played a racing game that was configured to induce low (Easy) or high (Hard) levels of demand. Both Easy and Hard levels of game demand were played with or without the presence of experimental pain using the cold pressor test protocol. Eight channels of fNIRS data were recorded from a montage of frontal and central-parietal sites located on the midline. Features were generated from these data, a subset of which were selected for classification using the RELIEFF method. Classifiers for game demand (Easy vs. Hard) and pain (pain vs. no-pain) were developed using five methods: Support Vector Machine (SVM), k-Nearest Neighbour (kNN), Naive Bayes (NB) and Random Forest (RF). These models were validated using a ten fold cross-validation procedure. The SVM approach using features derived from fNIRS was the only method that classified game demand at higher than chance levels (accuracy = 0.66, F1 = 0.68). It was not possible to classify pain vs. no-pain at higher than chance level. The results demonstrate the viability of utilising fNIRS data to classify levels of game demand and the difficulty of classifying pain when another task is present.

## Introduction

Awareness of pain and the ability to tolerate pain are influenced by selective attention (Torta et al., 2017). When attention is directed toward painful stimulation, awareness intensifies and pain tolerance declines (Bantick et al., 2002; Chayadi and McConnell, 2019). Conversely, when attention is distracted by a task unrelated to pain, the perceived intensity of pain declines and increased connectivity is observed between the default mode network and periaqueductal grey area (Kucyi et al., 2013). The influence of attention on the experience of pain is explained by a neurocognitive model with supporting evidence from neuroimaging research (Legrain et al., 2009, 2012).

This model of attention and pain can be exploited therapeutically by employing distraction to mitigate patients' experience of pain (Koller and Goldman, 2012; Williams and Ishimine, 2016). Neuroimaging research has demonstrated that distracting stimuli reduce activation in areas associated with acute pain, e.g., thalamus, somatosensory cortices, insula, anterior cingulate cortex (Birnie et al., 2017). Distraction techniques are deployed within paediatric medicine in both active and passive forms (Nilsson et al., 2013); the former corresponds to active engagement with task-related activity, such as a computer game, whereas the latter describes simply looking at pictures or watching a cartoon. The available evidence indicates that active forms of distraction are most effective (Wohlheiter and Dahlquist, 2013; Inan and Inal, 2019), presumably due to engagement of top-down attentional regulation that prioritises goal-related task stimuli over the bottom-up attentional processes triggered by the presence of pain (Legrain et al., 2009).

Technology is a highly effective form of distraction from pain, particularly Virtual Reality (VR) (Morris et al., 2009; Malloy and Milling, 2010; Trost et al., 2021) and computer games (Raudenbush et al., 2009; Jameson et al., 2011; Law et al., 2011). Technology distracts from painful stimulation by inducing an intense state of top-down attentional regulation in the player, which has been called immersion (Jennett et al., 2008) and flow (Csikszentmihalyi, 1990)—see Michailidis et al. (2018) for a discussion of both terms. For a game to induce a state of flow, it is important to consider how the skill level of the player must be balanced by the demand posed by the game, see Keller and Landhäußer (2012) for summary. Top-down mechanisms of attentional engagement are fully engaged and distraction from pain is maximal when: (a) challenge posed by the task can be matched by the skills of the person, (b) task goals are desirable, and (c) feedback on performance is present in an unambiguous form (Cowley et al., 2019). A systematic series of studies demonstrated that pain tolerance for experimental pain was found to increase in a linear fashion with game difficulty due to increased immersion (Fairclough et al., 2020). However, the need to sustain balance between challenge and skill places inherent limits on this relationship. Numerous studies (Richter et al., 2016) have demonstrated that task-related effort will decline if the person perceives success likelihood to be low, and this phenomenon has implications for the ability of a game to distract from pain. For example, perceived pain intensity decreased with increased game demand when patients in a burns clinic played a racing game, however, when game demand was increased to a level beyond the skill levels of the patients, their ratings of pain intensity reverted to baseline/no-game levels (Poole et al., 2014). This study illustrates the problem of using computer games to mitigate painful experiences. If the individual perceives the degree of challenge to be too easy or too difficult, top-down attentional engagement will erode and the game fails to function as an active distraction from pain. For a computer game to reliably mitigate the experience of pain, the game must adapt the level of demand to the skill level of the individual player, e.g., dynamic difficulty adjustment (DDA) (Zohaib, 2018). This type of personalised gaming experience can be achieved by a closed-loop approach to neuroadaptive gaming, wherein neurophysiological measures are collected (Liu et al., 2009; Ewing et al., 2016; Fernández et al., 2017) and analysed to create a real-time model of player state, which is subsequently used to dynamically adjust game demand to match the skills of the individual player.

The objective of the current paper is to assess the sensitivity of fNIRS (functional Near-InfraRed Spectroscopy) to monitor attentional engagement with game demand and the presence of pain. fNIRS has been selected as a potential measure for neuroadaptive gaming in this context for two reasons, firstly, earlier studies have demonstrated that neurovascular activation, particularly in the prefrontal cortex, is sensitive to increased demand during cognitive tasks, specifically: working memory load (Baker et al., 2018; Meidenbauer et al., 2021), increased task difficulty (Causse et al., 2017), and flow states when playing computer games (Harmat et al., 2015; de Sampaio Barros et al., 2018). Machine learning approaches have been utilised to classify levels of cognitive demand using laboratory-based (Naseer et al., 2016; Lu et al., 2020) and real-world tasks (Gateau et al., 2015; Verdière et al., 2018; Benerradi et al., 2019). There is also evidence of superior classification performance when using deep learning techniques (e.g., convolutional/artificial neural networks) to analyse fNIRS data in this context compared to classic machine learning approaches (e.g., *k*-Nearest Neighbours, Linear Discriminant Models, Support Vector Machine) (Naseer et al., 2016; Benerradi et al., 2019). Nevertheless, other studies have also reported superior classification performance using Support Vector Machines (SVM) (Lu et al., 2020) and Shrinkage Linear Discriminant Analysis (LDA) (Verdière et al., 2018), hence there is no clear consensus in the existing research literature. If fNIRS can distinguish periods of low from high attentional engagement in real-time, the resulting classification can be used to adjust the game difficulty upwards or downwards to sustain a state of flow or immersion.

fNIRS has also been used to measure cortical activation in response to painful stimulation using both clinical (Gentile et al., 2020) and non-clinical (Bandeira et al., 2019) groups. A small number of studies have applied machine learning techniques to fNIRS data in order to detect the presence of pain. A number of classification techniques were used to distinguish no-pain control from electrical pain stimulation using fNIRS data from prefrontal cortex (Lopez-Martinez et al., 2019), revealing superiority for a hierarchical Bayesian logistic regression compared to SVM. Fernandez Rojas et al. (2019) used fNIRS data to distinguish four types of sensory pain stimulations and found SVM to deliver high accuracy as a biomarker of pain, e.g., >89%. While these studies demonstrate the sensitivity of fNIRS to painful stimulation, it should be noted that classification accuracies were optimised by using data from a “pure” comparison between a rest condition and painful stimulation, i.e., no other sensory or cognitive stimulation is present except for the introduction of painful stimuli; therefore, it is uncertain whether fNIRS-based classification of pain would be viable in the presence of another active task, such as playing a game or any other simultaneous cognitive activity. If an acceptable classification of pain can be achieved while the person is engaged with a game, a neuroadaptive game using fNIRS would be able to assess the status of the player with reference to painful experience in addition to high/low engagement with the game.

An experiment was designed to explore the application of machine learning techniques to fNIRS data in order to: (1) classify levels of game demand, and (2) distinguish the presence of experimental pain from a non-pain condition while participants are engaged in a concurrent cognitive activity. The context for our experiment is a real-world application, i.e., development of a neuroadaptive gaming application for use in a pain clinic, hence we have deployed a sparse fNIRS montage, with respect to the number of optodes, to minimise the implementation requirements of a system that is to be used in a clinic. As a secondary goal, we wish to explore the sensitivity of fNIRS with reference to another category of measurement with a lower level of intrusiveness as a point of comparison; therefore, we also captured data from an electrocardiogram (ECG) because heart rate and heart rate variability can also be sensitive to cognitive demand (Forte et al., 2019) and pain (Koenig et al., 2014). The sensitivity of features derived from fNIRS and ECG to game demand and experimental pain were assessed as 20 participants played a racing game at two levels of demand (Easy, Hard), with and without the cold pressor test. The study utilised the RELIEFF algorithm (Kononenko et al., 1997) as a process of feature selection, which has been used in previous fNIRS research (Biswas et al., 2017; Aydin, 2020). The resulting features were used to classify game demand and pain using leave one out cross validation in combination with supervised learning from four machine learning techniques, namely, Support Vector Machine (SVM), k-Nearest Neighbour (kNN), naïve Bayes (NB), and Random Forest (RF).

## Method

### Participants

Data were collected from 20 participants (including 6 participants identifying as females) who were aged between 19 and 29 years (M = 22.75, SD = 3.23). Exclusion criteria included any history of cardiovascular disease, fainting, seizures, chronic or current pain, Reynaud's disease, or diabetes. Participants who were pregnant or had fractures, open cuts or sores on the feet or calves were also excluded. Approval for the study was obtained from the institutional research ethics committee prior to data collection. All participants were provided with a detailed Participant Information Sheet and provided written consent in advance of data collection.

### Design

The study was conducted as a within-participants design. All participants were exposed to two levels of game demand (Easy, Hard), under two conditions—with or without experimental pain via the cold-pressor test (CPT). As such, there were four conditions in the experimental design: Easy game, Hard game, Easy game + CPT, Hard game + CPT. The presentation order of all four conditions was counterbalanced across participants.

### fNIRS Recording

**Figure 1:**
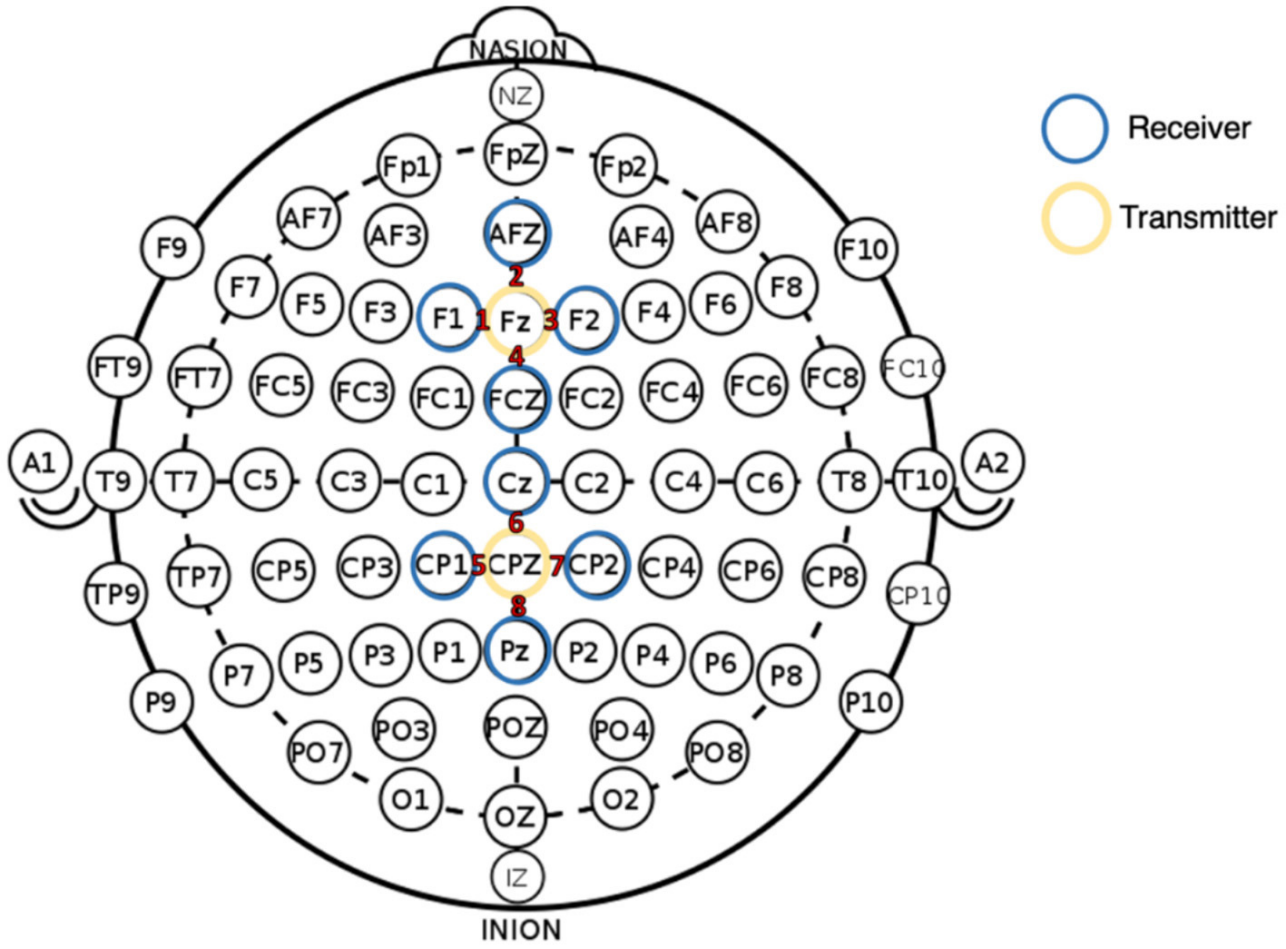
fNIRS montage with 2 sources (yellow circled) and 8 detectors (blue circled), illustrated with reference to the International 10–20 system (Jasper, 1958). All channel numbers are indicated in red text.

fNIRS data were collected using an Artinis Oxymon Mk III device using a montage that included two sources and eight detectors. Four channels were created between one source (Fz) and four detectors (F1, AFZ, F2, and FCz). This four-channel configuration was repeated at central-parietal sites, with a source at CPz and four detectors (CP1, Cz, CP2, and Pz) (see Figure 1). Table 1 provides the Montreal Neurological Institute (MNI) coordinates for each source-detector pair [48]. Source optodes emitted light at 847 and 761 nm wavelengths, with an inter-optode distance that varied between 2.85 and 3.90 cm when calculated as a Euclidean distance (see Table 1). The device was configured to sample at 10 Hz, and the Oxysoft data recording software (Artinis) was used for data capture.

**Table 1:** MNI coordinates for the fNIRS montage used during the study with approximate inter optode distances calculated as euclidean distance (cm).

		**x**	**y**	**z**	**Distance**
Channel 1	Transmitter–Fz	0.312	58.512	66.462	3.82
	Receiver–AFZ	0.231	80.771	35.417	
Channel 2	Transmitter–Fz	0.312	58.512	66.462	3.02
	Receiver–F2	29.514	57.602	59.540	
Channel 3	Transmitter–Fz	0.312	58.512	66.462	3.82
	Receiver–FCz	0.376	27.390	88.668	
Channel 4	Transmitter–Fz	0.312	58.512	66.462	2.85
	Receiver–F1	−27.495	56.931	60.342	
Channel 5	Transmitter–CPZ	0.386	−47.318	99.432	3.82
	Receiver–Cz	0.401	−9.167	100.244	
Channel 6	Transmitter–CPZ	0.386	−47.318	99.432	3.90
	Receiver–CP2	38.384	−47.073	90.695	
Channel 7	Transmitter–CPZ	0.386	−47.318	99.432	3.77
	Receiver–Pz	0.325	−81.115	82.615	
Channel 8	Transmitter–CPZ	0.386	−47.318	99.432	3.68
	Receiver–CP1	−35.515	−47.292	91.315	

### Accelerometer

A Shimmer3™ inertial measurement unit (IMU) was used to record head movement data via a three-axis accelerometer. This accelerometer data was used to remove the effects of head movement from the fNIRS signal (see section Procedure). The IMU had a sampling rate of 512 Hz and was worn over the fNIRS cap using an elasticated band. The Shimmer3^TM^ band was positioned around the rear and centre of the head, with the accelerometer device located just above the inion.

### Electrocardiogram

Raw electrocardiogram (ECG) was collected using a Zephyr BioHarness device, which collected data at a sampling rate of 250 Hz. This device was fitted to an elasticated strap and was worn by the participant under their clothing at the centre of their chest.

### Cold Pressor Test

A bespoke device was created (Dancer Designs) to administer the Cold Pressor Test (CPT). This device consisted of two water tanks, pumps, and a thermostat. One tank was designed for immersion of a limb (in this case, the foot to the depth of the ankle) during the test. The temperature of the water in the immersion tank was regulated via a negative control loop to sustain a constant temperature of 2°C. The purpose of the second tank was to “feed” the immersion tank with cold water in order that temperature in the latter was sustained at a constant temperature. Participants were instructed to place a foot in the immersion tank until the sensation of pain was too uncomfortable to bear. A stopwatch was used to record the amount of time that the foot remained immersed in the water.

This procedure was repeated for both levels of game demand experienced by participants in the game + pain condition. It should be noted that: (1) left and right feet were both used, allowing participants to alternate limbs between consecutive tests, (2) on completion of the cold pressor test, participants immersed the affected limb in a bowl of room temperature water to help restore normal circulation, and (3) due to the protocol (see Procedure section), there was an interval of at least 8 min between each application of the CPT to the same foot.

### Racing Game

**Figure 2:**
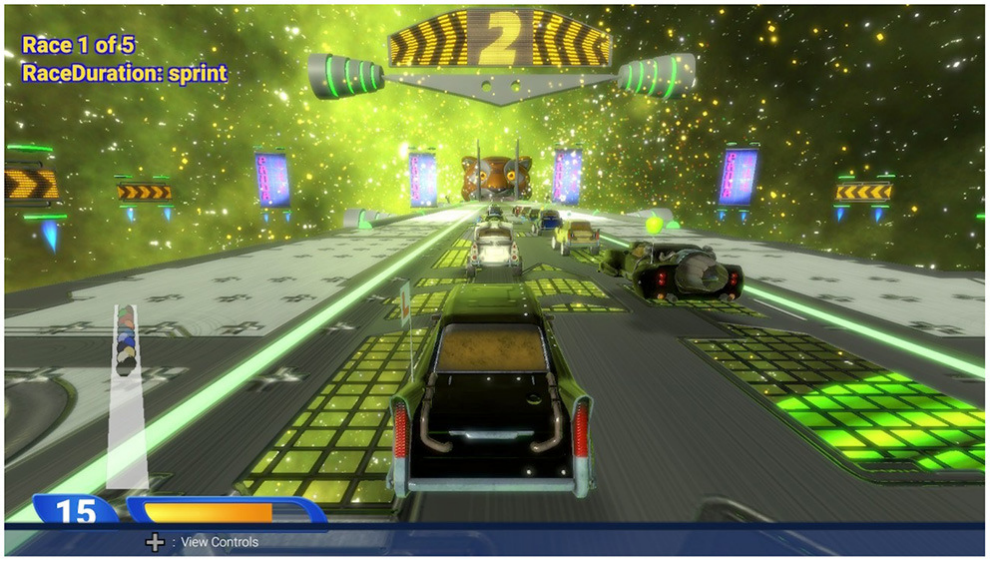
Screenshot of the racing game used in the study.

A racing game called “Space Ribbon” (Onteca Ltd.) was used for this study, which ran on a MacBook Pro. The game involved racing against computer-controlled opponents on a twisting track that floated in space (see Figure 2). The goal of the game was to win by achieving first position in the race, each of which lasted for ~180 s. Participants had at least three routes to winning the race: (1) superior handling of the vehicle, (2) using the slipstream of opponents to boost speed, and (3) pick up weapons that could be activated to temporarily disable opponents. Control of the vehicle during the game was achieved via a Sony PlayStation controller. Participants used the left joystick to control the position of the vehicle and the “X” button to accelerate.

Two levels of game difficulty were created in Space Ribbon for the purpose of experimentation, which were Easy and Hard. Game demand was manipulated by a combination of variables: (i) increasing the number of computerised opponents, (ii) enhancing the AI of the opponents (i.e., more efficient manoeuvring and strategic use of weapons), (iii) increasing the speed of all vehicles in the race, and (iv) increasing the manoeuvre speed of all vehicles. It was expected that all participants would easily achieve and maintain 1st place position during the Easy condition, whereas achieving a finishing position in the top four was highly unlikely during the Hard condition. A pilot test was performed (*N* = 5) whereby participants provided a self-reported ranking of game difficulty to ensure that the lowest ranking was associated with the Easy settings and the highest ranking was associated with the Hard settings.

### Subjective Questionnaires

Several questionnaires were included in the study to measure mental workload, motivation, and subjective immersion. The NASA Task Load Index (TLX) was used as a measure of subjective mental workload and consisted of six questions relating to mental demand, physical demand, temporal demand, performance, effort, and frustration (Hart and Staveland, 1988). The Motivation scale included eight questions and was derived from the Dundee State Stress Questionnaire (DSSQ) (Matthews, 2016). The final questionnaire was the Immersive Experience Questionnaire (IEQ), which was used as a measure of subjective immersion. The IEQ consisted of a 32-item questionnaire that related to aspects of gameplay, such as: attentional engagement, immersive experience, and enjoyment (Jennett et al., 2008). The IEQ, TLX, and Motivation scales were completed after each of the four experimental conditions.

### Procedure

On arrival at the laboratory, each participant was required to read an information sheet that explained the experiment procedures prior to signing a consent form. After consent was obtained, participants experienced the cold pressor test procedure purely as a familiarisation exercise, i.e., no data were recorded. They were subsequently required to perform a demo race of the Space Ribbon game to familiarise themselves with the game mechanics and associated controls. After familiarisation with the cold pressor test and game controls, participants were fitted with the BioHarness device, and the electrocardiogram signal was checked by the experimenter. The next phase of the protocol involved fitting the head cap and Oxymon sensors to the head of the participant. Once the fNIRS device had been attached and signal quality checked, the experimenter attached the accelerometer device to the headcap.

**Figure 3:**
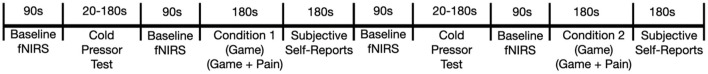
Timeline of procedure.

Participants experienced each level of game demand in accordance with the same procedure (see Figure 3): (1) a 90-s baseline where participant sat with eyes open, (2) a cold pressor test with no game, (3) a 90-s baseline, (4) Condition 1 where Easy or Hard game played with or without cold pressor test, (5) complete subjective questionnaires—and then this cycle is repeated for Condition 2, i.e., Easy or Hard game played with or without cold pressor test. The same cycle shown in Figure 3 was repeated for Conditions 3 and 4.

The presentation order of all four conditions was counterbalanced across participants. Half of the participants experienced the Easy game with and without the cold pressor test as their first condition followed by the Hard game and vice versa. The presentation of Game and Game + Pain conditions were counterbalanced within each level of game demand. With respect to those games where participants experienced the cold pressor test, participants were instructed to place their foot in the immersion tank during the countdown at the beginning of each game. When the sensation of pain was too uncomfortable to bear, they removed their foot from the water and continued to play until the end of the race. When the participants had completed all four experimental conditions, the fNIRS and other sensors were removed, they were thanked for their participation and debriefed.

### Signal Processing

The raw ECG signal from the BioHarness was processed and corrected for artefacts using a bespoke signal processing algorithm developed within our institution. For a detailed description, see Dobbins and Fairclough (2018). After artefacts were identified and corrected, the algorithm calculated heart rate (HR) from the clean signal.

Several philtres and algorithms were also applied to the fNIRS data that were developed as a bespoke signal processing pipeline in MATLAB. In order to determine neurovascular activation in the target cortices, Optical Density data were converted into levels of oxygenated haemoglobin (HbO) and deoxygenated haemoglobin (Hbb), using the modified Beer Lambert Law (mBLL) (Baker et al., 2014). These data were subsequently filtered using a 6th Order Chebyshev philtre, with passband edge frequencies of 0.09 and 0.01 Hz for low and high pass filtering; for discussion on selection of cut-off bands for fNIRS filtering, see Pinti et al. (2019).

Both fNIRS and accelerometer data were processed using the Acceleration-Based Movement Artefact Reduction Algorithm (AMARA) (Metz et al., 2015). AMARA detects periods of movement within an accelerometer signal and then compares these periods of movement to the fNIRS data. If the moving standard deviation (MSD) from the fNIRS signal has changed significantly during the same period that movement has been detected within the accelerometer signal, these segments of fNIRS data are marked as artefact segments. Reconstruction of artefact segments uses forward and backward baseline adjustments and interpolation to reconstruct the entire signal to correct for movement artefacts. After the application of the AMARA algorithm, the Correlation Based Signal Improvement (CBSI) algorithm (Cui et al., 2010) was applied to the data. The application of CBSI effectively renders HbO and Hbb negatively correlated and only HbO was subsequently used in the analyses.

### Feature Extraction

A number of statistical heart rate features were extracted from the processed ECG signal, which were calculated across windows of 8-s and included: average, median, maximum, minimum, range, and standard deviation.

Features for HbO were also extracted from the 8-s analysis windows. This 8-s window was selected because the haemodynamic response takes ~7-s to reach its maximum amplitude (Kamran et al., 2016). fNIRS data were recorded continuously throughout both Easy and Hard games, yielding a maximum of 23 epochs, i.e., 180 s (total duration of each game)/8 s (time window for calculation of features). These 23 epochs were utilised in the classification of Easy vs. Hard game demand alongside 23 epochs of ECG data.

A number of descriptive statistics were generated for HbO based on the calculations described in Verdière et al. (2018), including: Mean, Peak, Skew, Variance, and Area Under the Curve (AUC). As the eight channels of the montage were split evenly between the Prefrontal and Somatosensory cortex, associations between the different channels of HbO have also been investigated. Connectivity features were extracted based on 28 possible connexions between the eight recorded channels, e.g., Ch1 × Ch2, Ch1 × Ch3, Ch1 × Ch4, Ch1 × Ch5 etc. Two measures of connectivity were calculated for every 8 s window, including Pearson's Correlation Coefficient and Wavelet Coherence. Pearson's Correlation Coefficient (see equation 1) is the covariance for two signals, normalised by their standard deviation. In this equation, x = the values from the first channel (i.e., Ch1) and y = the values from the second channel (i.e., Ch2).


(1) $$\begin{array}{l} Pearson\left(x,y\right)=\frac{COV\left(x,y\right)}{std{\left(x\right)}^{*}std\left(y\right)} \end{array}$$

Values from wavelet coherence (see equation 2) were averaged for frequencies between 0.3125 and 0.08 Hz, as recommended by the fNIRS literature (Cui et al., 2012; Verdière et al., 2018). In this equation, S = fNIRS signal, W = wavelet, and x and y = fNIRS channels (i.e., Ch1 and Ch2) respectively.


(2) $$R_{n}^{2}\left(s\right)=\frac{|S(s^{-1}W_{n}^{xy}{\;(s)|}^{2}}{S\left(s^{-1}{\left|W_{n}^{x}\left(s\right)\right|}^{2}\right)S\left(s^{-1}{\left|W_{n}^{y}\left(s\right)\right|}^{2}\right)}$$

To create datasets for classification of pain vs. no-pain condition in both Easy and Hard demand conditions, we required two data files of the same duration at each level of game demand. This was a challenge because the duration of the game + pain condition (i.e., the maximum duration that the participant kept the foot in the cold water) varied across participants and game demand. Therefore, it was necessary to reduce the size of the data file from the no-pain/game only condition to match the maximum duration that was achieved during the game + pain condition, both for each level of demand and for each individual participant. This procedure ensured that data for the pain vs. no-pain comparison accurately represented a contrast between playing the game with and without painful stimulation and that the resulting datasets were balanced.

However, by adopting this technique, we generated imbalanced labelled datasets for Heart Rate data, i.e., the no-pain condition was the majority class. Classification of unbalanced data can lead to bias and incorrect accuracy, as classifiers are more likely to detect the majority class. A common approach to address imbalance is to re-sample the dataset by either randomly under-sampling the majority class, which reduces the dataset and runs the risk of losing important data, or oversampling the minority class, which can lead to overfitting (Maimon and Rokach, 2010). As the dataset was already restricted in size, the Heart Rate data was oversampled to ensure there were an even number of datapoints for each of the binary categories. The data were randomly oversampled using the common Synthetic Minority Over-sampling Technique (SMOTE) (Chawla et al., 2002; Fernández et al., 2018).

### Feature Selection

To reduce the feature space prior to classification, feature selection was undertaken using the RELIEFF algorithm (Kononenko et al., 1997). This algorithm was chosen for its efficiency and computation simplicity (Urbanowicz et al., 2018), as well as its usage in fNIRS based BCI research (Aydin, 2020). The RELIEFF algorithm uses a *k* nearest neighbour approach to calculate a weight for each feature, which represents its ability to distinguish samples between classes and evaluate the quality of each feature (e.g., Chen et al., 2019). The *k* value determines the number of nearest neighbours in relation to the number of nearest hits (i.e., nearest values in the same class) and misses (i.e., nearest values in a different class) per class. The resulting feature weights are sorted in descending order where the feature with the largest weight is ranked first. The weighted features are then plotted on a graph, and a cut-off point to select the most relevant features for classification is determined by the “elbow” of this graph, i.e., the point at which the feature weights markedly declines), see Wosiak and Zakrzewska (2018) for example, and Urbanowicz et al. (2018) for further discussion.

Classification models were constructed using the fNIRS and HR features to determine, (1) the demand of the game that the participant was playing (i.e., Easy vs. Hard), (2) if the participant was experiencing pain whilst playing the game during the Easy (Pain/No Pain) and (3) Hard (Pain/No Pain) levels of game demand.

The process of feature selection was undertaken using MATLAB version R2021a and undertaken independently for each of these classification conditions. Each dataset was firstly split into training and testing sets. Using a random selection of participants, 60% of the participants were used for training and the remaining 40% for testing. The RELIEFF algorithm was then applied to the training sets. The value of *k* to use within RELIEFF was determined by calculating the square root of the total number of features. If this calculation resulted in an even number, *k* was increased by one to ensure it was odd, which is common practise when dealing with a nearest neighbour approach (Islam et al., 2007). The *k*-value varied depending on which dataset was used: all fNIRS datasets used a *k-*value of 15. The process of randomly splitting the dataset and applying RELIEFF was repeated ten times, for each dataset, to ensure the generalisability of the results.

**Figure 4:**
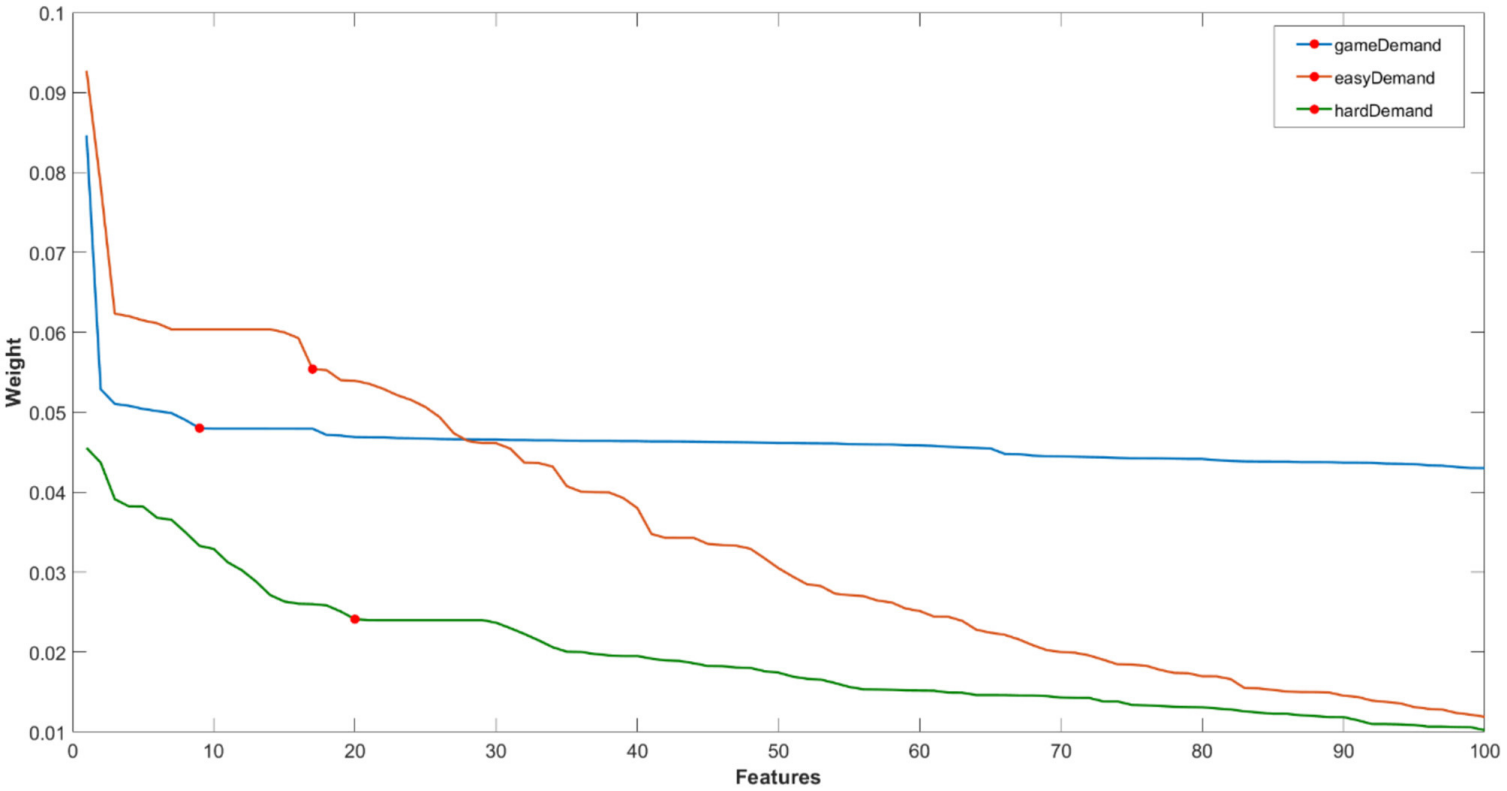
Example of one loop of ranked feature weights from RELIEFF algorithm for feature selection for the classification of fNIRS features of (i) game demand (easy vs. hard), (ii) easy game demand, and (iii) hard game demand (pain vs. no pain) using the fNIRS features. Red circle = feature cut-off points for each classification problem.

For ease of visualisation, an example of one loop of the first 100 ranked features for each classification problem [Game Demand (Easy vs. Hard), Easy game demand and Hard game demand (Pain vs. No Pain)] have been plotted in Figure 4. Features were selected with reference to the “elbow” in the data where the weights trail off (i.e., the marked red circles in the graph).

**Figure 5:**
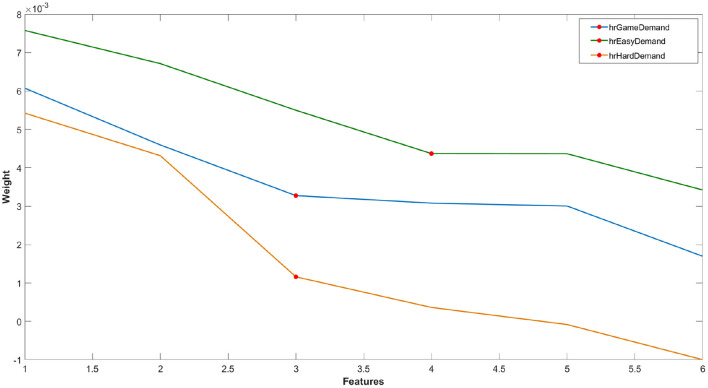
Example of one loop of ranked feature weights from RELIEFF algorithm for feature selection for the classification of HR features of (i) game demand (easy vs. hard), (ii) easy game demand and (iii) hard game demand (pain vs. no pain). Red circle = feature cut-off points for each classification problem.

For reasons of parity, the RELIEFF algorithm was also applied to the six features of the HR data using the same process described above for each classification model (Figure 5). All HR datasets used a *k* value of 3.

### Classification

The process of classification consisted of comparing parametric and non-parametric classification algorithms to determine which produced the highest accuracy for classification of Easy/Hard game demand and pain/no-pain. Four algorithms were selected, which were: Support Vector Machine (SVM), k-Nearest Neighbour (kNN), Naive Bayes (NB), and Random Forest (RF). These methods were selected on the basis of their previous applications for fNIRS-based BCI and classification of experimental pain (Naseer and Hong, 2015; Hong et al., 2018; Fernandez Rojas et al., 2019; Kwon et al., 2020).

**Figure 6:**
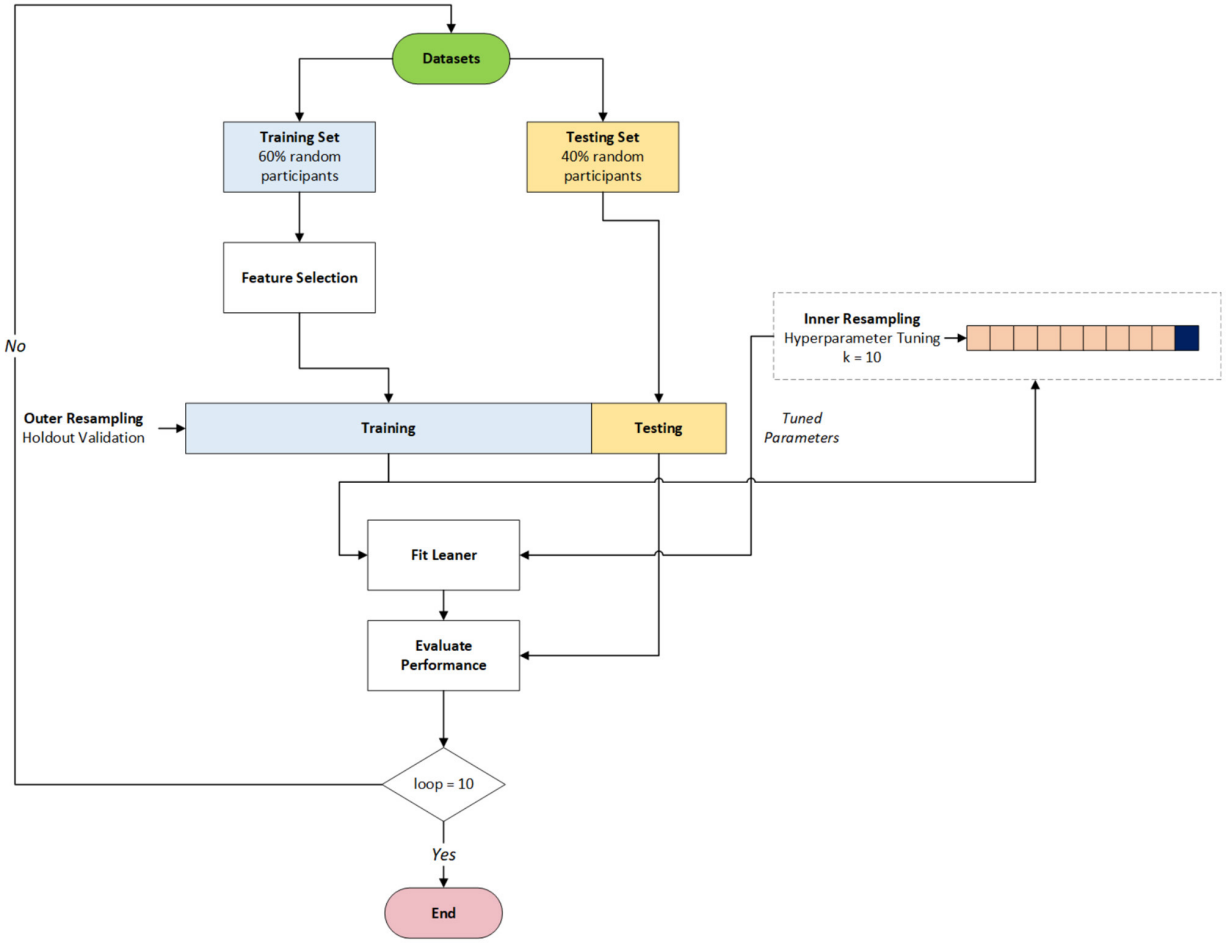
Cross-Validation approach.

Classification was undertaken using R version 3.6.3 and the mlr3 package (Lang et al., 2019). In order to obtain an unbiased estimate of performance, the classification models were constructed using a nested cross-validation approach that contains an inner and outer resampling loop (Becker, 2021) (see Figure 6).

Following the approach set out in Becker (2021) and using the selected RELIEFF features, the outer resampling loop utilises a holdout validation approach using the same training/testing sets that were defined during the feature selection stage. Using the training set of the outer loop, hyperparameter tuning was performed by triggering the inner resampling loop, which used *k*-fold cross-validation (*k* = 10) and the grid search tuning method. In this way, each outer training set generates one group of designated hyperparameters. Each classification algorithm is then fitted on each outer training set using the selected hyperparameters (Becker, 2021). The performance of each algorithm is then evaluated using the outer test sets. In order to provide generalisability, this process was repeated ten times for each classification algorithm (note: the tuning inner loop was not performed on NB due to the simplicity of the algorithm). Information regarding the tuned hyperparameters for each classification can be found within the Appendices section of this paper.

Each classification method (SVM, kNN, NB, and RF) was utilised to classify Easy from Hard level of game difficulty using the selected features from the fNIRS data (see Figure 4). This analysis was subsequently repeated using selected features from heart rate (see Figure 5). The classification of pain vs. no-pain was conducted using all four methods in two separate analyses, one for Easy game demand and a second using data from Hard demand game. The fNIRS features selected for both analyses are listed in Figure 4, and once again, both analyses were repeated using selected heart rate features (see Figure 5).

The level of game demand was classified using two sets of features, one derived from fNIRS (Figure 4) and a second analysis using heart rate features (Figure 5). Four indices of performance are reported for each classification, each one is represented by the mean and standard deviation across all ten loops:

- Accuracy—proportion of true positives and true negatives from all classifications, calculated as an average
- F1—the harmonic mean of precision and recall, which represents a robust measure of correctly classified cases
- True Positive Rate (sensitivity)—the proportion of true positives from all classifications
- True Negative Rate (specificity)—the proportion of true negatives from all classifications
- Area Under Curve (AUC)—the area under the ROC (Receiver Operating Characteristic) curve, which is a plot of FPR vs. TPR at different classification thresholds.

## Results

### Behavioural and Subjective Data

The CPT time is the total duration (secs) that participants kept their limb immersed in the cold water and this variable was used as a behavioural measure of pain tolerance. These data were subjected to a univariate ANOVA (baseline/no game vs. Easy demand vs. Hard demand). The baseline data was obtained by averaging across all four baselines that participants performed during the experimental protocol. Two participants who kept their foot in the water for the maximum duration of 180 s in all three conditions were omitted from this analysis. This model revealed a significant main effect [*F*_(2, 16)_ = 9.91, *p* = 0.05, eta^2^ = 0.55], Bonferroni tests revealed that baseline CPT times (M = 22.5 s, sd = 9.75) were significantly lower than either of the two game demand conditions (*p* < 0.01), but there was no significant difference between Easy (M = 48.5 s, sd = 35.73) and Hard (M = 74.07 s, sd = 64.11) levels of game demand (*p* = 0.08).

As a manipulation cheque, we collected subjective measures of mental workload (TLX), immersion (IEQ), and motivation (DSSQ). Descriptive statistics are provided in Table 2. All three scales were subjected to the same 2 (pain/no pain) × 2 (Easy/Hard demand) ANOVA. The analyses of subjective workload revealed a significant main effect for demand [*F*_(1, 18)_ = 23.51, *p* < 0.01, eta^2^ = 0.56] but there was no significant effect of pain [*F*_(1, 18)_ = 0.17, *p* = 0.68]. There was a significant effect of game demand on subjective immersion [*F*_(1, 18)_ = 12.42, *p* < 0.01, eta^2^ = 0.40] with participants reporting increased immersion at high game demand (see Table 2), but no equivalent main effect of pain [*F*_(1, 18)_ = 1.71, *p* = 0.21]. The analyses of subjective motivation revealed a significant interaction between pain and game demand [*F*_(1, 18)_ = 4.12, *p* = 0.05, eta^2^ = 0.18]; *post-hoc t*-tests indicated that subjective motivation was significantly higher during the game + pain compared to the game only condition, but this effect was only observed when game demand was Easy [*t*_(19)_ = 2.01, *p* = 0.01].

**Table 2:** Descriptive statistics (mean with standard deviations in parentheses) for subjective measures of mental workload, immersion, and motivation across all conditions (*N* = 20).

	**No pain**		**Pain**	
	**Easy**	**Hard**	**Easy**	**Hard**
Workload (TLX)	3.15 [1.25]	4.60 [1.52]	3.31 [1.24]	4.58 [1.22]
Immersion (IEQ)	126.35 [31.34]	152.10 [19.96]	133.10 [32.96]	152.45 [19.33]
Motivation	38.20 [6.09]	40.85 [7.02]	39.60 [5.76]	40.10 [5.45]

### Classification of Game Demand

The level of game demand was classified using two sets of features, one derived from fNIRS (Figure 4) and a second analysis using heart rate features (Figure 5). Four indices of performance are reported for each classification, each one is represented by the mean and standard deviation across all ten folds of the cross-validation. The mean classification performance across all ten folds for Easy vs. Hard levels of Game Demand using the fNIRS features are illustrated in Table 3, whilst Table 4 depicts the corresponding Heart Rate feature results. The tuned hyperparameters for the results depicted in Table 3 can be found within Appendix 1, whilst the corresponding parameters associated with Table 4 can be found in Appendix 2.

**Table 3:** Mean classification performance for easy vs. hard levels of game demand using fNIRS features across all ten folds, standard deviations in parentheses.

	**NB**	**kNN**	**RF**	**SVM**
Accuracy	0.429 [0.07]	0.576 [0.07]	0.544 [0.09]	0.664 [0.07]
F1	0.422 [0.08]	0.563 [0.08]	0.526 [0.09]	0.683 [0.08]
True positive rate	0.426 [0.12]	0.554 [0.12]	0.513 [0.13]	0.731 [0.12]
True negative rate	0.431 [0.16]	0.599 [0.16]	0.576 [0.21]	0.598 [0.11]
AUC	0.383 [0.10]	0.614 [0.07]	0.580 [0.07]	0.704 [0.07]

*NB, Naïve bayes; kNN, k-nearest neighbour; RF, Random forest; SVM, Support vector machine*.

**Table 4:** Mean classification performance for easy vs. hard levels of game demand using heart rate features across all ten folds, standard deviations in parentheses.

	**NB**	**kNN**	**RF**	**SVM**
Accuracy	0.539 [0.03]	0.504 [0.03]	0.502 [0.04]	0.533 [0.03]
F1	0.476 [0.10]	0.429 [0.05]	0.412 [0.05]	0.356 [0.11]
True positive rate	0.467 [0.06]	0.404 [0.07]	0.375 [0.04]	0.118 [0.17]
True negative rate	0.598 [0.09]	0.594 [0.09]	0.614 [0.06]	0.895 [0.15]
AUC	0.548 [0.04]	0.497 [0.06]	0.509 [0.04]	0.501 [0.05]

*NB, Naïve bayes; kNN, k-nearest neighbour; RF, Random forest; SVM, Support vector machine*.

An analysis of classification was performed using ANOVA models. The level of chance performance for binary classification is 0.5 from a theoretical perspective, however, this level should be corrected for sample size (Combrisson and Jerbi, 2015). If we adjust chance level for a sample size of 400 used for both classification analyses reported in Tables 3, 4, the corrected level of chance at *p* < 0.05 is 0.54. Therefore, we constructed a 3-way ANOVA model to compare levels of accuracy when classifying easy and hard game demand across all ten folds for features derived from fNIRS and HR, compared to adjusted level of chance (Combrisson and Jerbi, 2015); this model was applied to each classifier.

The analysis of accuracy using Naïve Bayes revealed a significant main effect [*F*_(2, 8)_ = 18.48, *p* < 0.01, eta^2^ = 0.82], Bonferroni contrasts indicated that accuracy was significantly lower for the NB classifier derived from fNIRS features compared to adjusted level of chance; there was no significant difference between accuracy using HR features compared to chance. The same analysis was applied to the k-Nearest Neighbour classifier. This analysis revealed a significant main effect [*F*_(2, 8)_ = 6.31, *p* = 0.023, eta^2^ = 0.61] wherein accuracy derived from HR features (0.504) was significantly lower than accuracy from fNIRS (0.576) or the adjusted chance level of 0.54. This pattern was repeated for the analyses of classification using the Random Forest classifier, but did not reach statistical significance [*F*_(2, 8)_ = 3.68, *p* = 0.07]. The analysis of classification performance using a Support Vector Machine revealed a significant effect [*F*_(2, 8)_ = 23.19, *p* < 0.01, eta^2^ = 0.85]. Bonferroni contrasts indicated that accuracy levels using fNIRS features (0.664) were significantly higher than either accuracy using HR features (0.533) or chance.

### Classification of Pain

The classification of participants playing the game with and without experimental pain was carried out via four analyses: (1) using selected features from fNIRS (Figure 4) when participants played a game at Easy demand, (2) using heart rate features when participants played the Easy game (Figure 5), (3) using selected fNIRS features (Figure 4) when participants played a game at Hard demand, and (4) using heart rate features when participants played the game at Hard demand (Figure 5). The results of the pain vs. no-pain classification using fNIRS features are presented in Table 5, the results of the same classification using heart rate features are provided in Table 6. The tuned hyperparameters for the Easy Demand results depicted in Table 5 can be found within Appendix 3, whilst Hard Demand results are in Appendix 4.

**Table 5:** Mean classification performance for pain vs. no-pain using selected fNIRS features during easy and hard levels of game demand across all ten folds, standard deviations in parentheses.

		**NB**	**kNN**	**RF**	**SVM**
Easy demand	Accuracy	0.543 [0.09]	0.560 [0.12]	0.544 [0.12]	0.536 [0.08]
	F1	0.466 [0.15]	0.572 [0.12]	0.542 [0.18]	0.534 [0.15]
	TPR	0.377 [0.22]	0.600 [0.17]	0.577 [0.23]	0.506 [0.26]
	TNR	0.709 [0.21]	0.520 [0.18]	0.511 [0.14]	0.566 [0.18]
	AUC	0.616 [0.11]	0.560 [0.12]	0.565 [0.13]	0.507 [0.11]
Hard demand	Accuracy	0.429 [0.16]	0.561 [0.09]	0.583 [0.11]	0.546 [0.11]
	F1	0.411 [0.15]	0.558 [0.14]	0.575 [0.16]	0.549 [0.12]
	TPR	0.397 [0.21]	0.580 [0.19]	0.603 [0.23]	0.546 [0.13]
	TNR	0.460 [0.15]	0.543 [0.07]	0.563 [0.10]	0.546 [0.14]
	AUC	0.478 [0.16]	0.570 [0.12]	0.592 [0.15]	0.569 [0.11]

*NB, Naïve bayes; kNN, k-nearest neighbour; RF, Random forest; SVM, Support vector machine; TPR = True Positive Rate, TNR = True Negative Rate*.

**Table 6:** Mean classification performance for pain vs. no-pain using selected heart rate features during easy and hard levels of game demand across all ten folds, standard deviations in parentheses.

		**NB**	**kNN**	**RF**	**SVM**
Easy demand	Accuracy	0.507 [0.06]	0.510 [0.04]	0.507 [0.05]	0.492 [0.05]
	F1	0.447 [0.10]	0.491 [0.04]	0.468 [0.06]	0.373 [0.19]
	TPR	0.457 [0.16]	0.523 [0.09]	0.475 [0.07]	0.283 [0.04]
	TNR	0.557 [0.08]	0.506 [0.12]	0.537 [0.09]	0.685 [0.39]
	AUC	0.525 [0.07]	0.515 [0.05]	0.509 [0.06]	0.454 [0.03]
Hard demand	Accuracy	0.503 [0.02]	0.528 [0.03]	0.514 [0.05]	0.504 [0.04]
	F1	0.472 [0.12]	0.496 [0.03]	0.483 [0.05]	0.354 [0.24]
	TPR	0.452 [0.24]	0.484 [0.05]	0.475 [0.07]	0.241 [0.35]
	TNR	0.562 [0.24]	0.570 [0.06]	0.551 [0.08]	0.769 [0.33]
	AUC	0.516 [0.04]	0.550 [0.03]	0.528 [0.06]	0.524 [0.05]

*NB, Naïve bayes; kNN, k-nearest neighbour; RF, Random forest; SVM, Support vector machine; TPR = True Positive Rate, TNR = True Negative Rate*.

The classification results for pain classification using heart rate features are reported in Table 6. The tuned hyperparameters for the Easy Demand results depicted in Table 6 can be found within Appendix 5, whilst Hard Demand results are in Appendix 6.

The classification accuracy data was analysed via the same ANOVA approach described in the previous section. In the case of all pain/no pain classifications, the sample size was reduced to 160, which yielded an adjusted rate of chance of 0.5625. The results of the analyses are described in Table 7.

**Table 7:** ANOVA results and summary from analyses of pain classifications for easy and hard levels of game demand.

	**Classifier**	***F*_(2,8)_**	***p***	**eta^**2**^**	**Significant effects**
Easy demand	NB	3.88	0.07	0.49	HR < chance
	kNN	7.28	0.02	0.65	
	RF	4.94	0.04	0.55	
	SVM	8.42	0.01	0.68	
Hard demand	NB	9.82	<0.01	0.40	HR and fNIRS < chance
	kNN	8.67	0.01	0.68	HR < chance
	RF	5.61	0.03	0.58	HR < chance & fNIRS
	SVM	9.38	<0.01	0.70	HR < chance

The ANOVA analyses revealed that classification of pain across both levels of game demand did not significantly exceed chance levels using fNIRS or HR features.

## Discussion

The goal of the current study was to assess whether game difficulty and the presence of experimental pain could be classified via implicit measures of neurophysiology (fNIRS) and psychophysiology (heart rate). Our manipulation of Easy/Hard game difficulty, which was selected via pilot testing (see section Cold pressor test) and self-report data (Table 2), confirmed that both mental workload and immersion were perceived as significantly higher during Hard compared to the Easy demand game. Pain tolerance was operationalised by calculating the total time of the cold pressor test for each participant. As expected, playing a game increased pain tolerance in comparison to a no-game control due to the attentional mechanisms of active distraction described in the introduction (Torta et al., 2017). However, pain tolerance did not significantly increase during the Hard game compared to the Easy game as observed in earlier studies (Fairclough et al., 2020). Closer inspection of cold pressor test times revealed an increase of 25 s during the Hard game compared to the Easy game, but with high variability, i.e., standard deviations were 35.73 and 64.11 s for Easy and Hard games, respectively. High-levels of inter-participant variability is a known problem for the cold pressor test, even when implemented with specialised apparatus and a standardised protocol (Von Baeyer et al., 2005). The presence of high variability in our cold pressor data meant that our within-participants comparison achieved a significance level of *p* = 0.08, which was above our alpha threshold of 5%.

A range of features were derived from the fNIRS (Figure 4) and HR (Figure 5) data, which were used to make a binary classification between Easy and Hard game demand. We utilised a nested resampling ten fold cross-validation method across a variety of classifiers (Tables 3, 4). The results illustrated that: (1) SVM yielded the highest level of accuracy (66.4%) from fNIRS features, (2) maximum accuracy for HR features was 53.9% using a NB classifier, and (3) only the SVM classifier using fNIRS features achieved a level of classification performance that was significantly above chance levels. This absolute level of accuracy is somewhat lower than similar laboratory-based, subject-independent, binary classifications of mental workload reported in earlier studies, e.g., 0.83 (Lu et al., 2020), 0.84 (Naseer et al., 2016), and applied tasks, e.g., 0.71 (Benerradi et al., 2019), 0.80 (Gateau et al., 2015); however, direct comparisons between the current study and related research are problematic, as earlier experiments manipulated workload using task simulation (e.g., aviation) or standardised laboratory tasks, as opposed to a computer game. In general, the classification of game demand using heart rate features compared poorly with features derived from fNIRS for all classifiers except NB, indicating the superiority of fNIRS features for classification of game demand, with the caveat that the number of features selected for the fNIRS-based model was significantly higher.

Our approach also utilised both HR and fNIRS features to classify the presence of pain within both Easy and Hard levels of demand (Tables 5, 6)—but none of the resulting models were able to discriminate game play in the presence of the cold pressor test from game play without the cold pressor test at an accuracy level above chance; in fact, classification based on HR features fell consistently and significantly below chance. Other researchers have utilised machine learning analyses based on fNIRS features to classify the presence of pain and reported high levels of accuracy. For example, Lopez-Martinez et al. (2019) reported classification accuracy of 0.69 using SVM, which improved to 0.81 with a non-parametric, hierarchical Bayesian Multi-Task Learning model, Fernandez Rojas et al. (2019) also reported high levels of accuracy when applying SVM-based classification to their pain data. There are several reasons why classification of pain did not achieve high accuracy during the current study in both relative (compared to other studies) and absolute (greater than chance) terms: (1) unlike Lopez-Martinez et al. (2019) and Fernandez Rojas et al. (2019), we measured experimental pain in conjunction with playing a computer game and it is very likely that variability introduced into the physiological data by the game play degraded classification accuracy for pain, (2) unlike earlier work, we utilised the cold pressor test as our method to induce experimental pain, which was not personalised to individual pain thresholds, hence there are enormous individual variability in the duration of pain induction and the experience of pain by individual participants, and (3) the cold pressor test delivers a cumulative experience of pain, i.e., pain increases in intensity over the period of immersion, hence pain may have varied significantly throughout the period of data collection labelled as pain. In addition, we did not distinguish between the sensory experience of placing one's foot in water (at room temperature) from the experience of immersing the foot in very cold water as part of the cold pressor test; therefore, the resulting fNIRS data is limited by our inability to distinguish a sensory experience from a painful sensory experience. This ambiguity, along with the absence of calibration, may also be responsible for the high level of inter-individual variability observed in our pain tolerance data. The current protocol could be improved by using a more precise and personalised approach to pain induction, such as the quantitative sensory testing (QST) method utilised by Fernandez Rojas et al. (2019).

With respect to limitations and improvements to the current protocol, the settings of the Easy and Hard levels of game demand were problematic. We performed a small pilot study using subjective self-report measures to establish Easy and Hard demand by manipulating key parameters of the game, as described in Section Racing game, which represented a “one-size-fits-all” approach to establishing levels of game demand. However, it would have been optimal to calibrate game demand (Xue et al., 2017; Sarkar and Cooper, 2019) to the skills of the individual participant, which would have increased the reliability of the Easy/Hard manipulation by striking the desired balance between player skill and objective game demand (Keller and Landhäußer, 2012).

The current study utilised fNIRS as a technique to measure attentional engagement as a response to increased game demand. It could be argued that our adoption of game difficulty as a “ground truth” for labelling classification data was circular, as we could have simply derived our classification labels directly from gaming parameters, but we would argue against this interpretation. Previous work (Ewing et al., 2016; Fairclough et al., 2018) demonstrated how task demand dissociates from cortical measures of attentional engagement when success likelihood is low, as predicted by motivational intensity theory (Richter et al., 2016). When the player is pushed to the limits of their skill level by game demand, engagement and objective task demand are no longer synonymous. Subjective self-report measures are popular choices for the derivation of classification labels, but this approach is problematic as self-report data are retrospective, susceptible to bias and difficult to administer *in situ* without interfering with gameplay (Burns and Fairclough, 2015). Others have argued for a multidimensional approach for the measurement of player engagement (Martey et al., 2014), which combines contextual data (such as game demand) with real-time measures from physiology and gameplay (Yannakakis et al., 2013) in order to model the state of the player. While a multidimensional approach offers definite advantages as strategy for accurate operationalisation, it also introduces complications for the derivation of unambiguous labels for supervised learning methods.

The analyses conducted in the current paper were representative of subject-independent classification using supervised learning in combination with classic machine learning techniques. This approach was adopted because the long-term objective of the current research is the development of a neuroadaptive game to be used in the clinic, hence we did not explore techniques that required generation of training dataset for each individual user prior to system usage, e.g., unsupervised, subject-dependent techniques. However, other researchers working with fNIRS data have reported superior classification with artificial neural networks (Naseer et al., 2016) and deep learning techniques, such as fully convolutional networks (Lu et al., 2020); the latter reported an accuracy level above 97% for subject-independent classification of cognitive demand with deep learning. A subject-independent, unsupervised approach could be explored in future work, especially if a large training dataset could be generated that produced a high level of subject-independent classification.

As the motivation of the study was to explore the viability of fNIRS neuroadaptive gaming concept, the current paper demonstrated that increased game demand can be detected at a level significantly higher than chance by applying SVM to features derived from fNIRS. This “live” model of the player could be used as an input to an adaptive gaming system, where implicit neurophysiological monitoring is used to index the response of the player to increased game demand. With respect to efficiency of set-up, comfort, and intrusiveness, heart rate measures would be preferable for this type of application in a clinic, however, our results indicated that classification of game demand based on heart rate features failed to exceed the level of chance.

## Data Availability Statement

The raw data supporting the conclusions of this article will be made available by the authors, without undue reservation.

## Ethics Statement

The studies involving human participants were reviewed and approved by University Research Ethics Committee (UREC) Liverpool John Moores University. The patients/participants provided their written informed consent to participate in this study.

## Author Contributions

SF contributed to the planning of the study, piloting and setting-up the study, performance of the data analyses, and was primary author of the manuscript. CD contributed to planning of the study, piloting and setting-up the study, data analyses, and reviewing the manuscript. KS contributed to planning of the study, conducting the pilot trials, experimental trials, performance of data analyses, drafting, and reviewing the manuscript. All authors contributed to the article and approved the submitted version.

## Funding

This work was funded internally by Liverpool John Moores University as a Ph.D., scholarship.

## Conflict of Interest

The authors declare that this study received support from Onteca Ltd who supplied the game used during the study, and an SDK that allowed the authors to manipulate the level of game demand. The company was not involved in the study design, collection, analysis, interpretation of data, the writing of this article or the decision to submit it for publication.

## Publisher's Note

All claims expressed in this article are solely those of the authors and do not necessarily represent those of their affiliated organizations, or those of the publisher, the editors and the reviewers. Any product that may be evaluated in this article, or claim that may be made by its manufacturer, is not guaranteed or endorsed by the publisher.

## References

[B1] Aydin E. A. (2020). Subject-Specific feature selection for near infrared spectroscopy based brain-computer interfaces. Comput. Methods Programs Biomed. 195:105535. 10.1016/j.cmpb.2020.105535 32534382

[B2] Baker J. M. Bruno J. L. Gundran A. Hadi Hosseini S. M. Reiss A. L. (2018). fNIRS measurement of cortical activation and functional connectivity during a visuospatial working memory task. PLoS ONE 13:e0203233. 10.1371/journal.pone.0203233 30071072 PMC6072025

[B3] Baker W. B. Parthasarathy A. B. Busch D. R. Mesquita R. C. Greenberg J. H. Yodh A. G. (2014). Modified beer-lambert law for blood flow. Biomed. Opt. Express 5, 4053–4075. 10.1364/BOE.5.004053 25426330 PMC4242038

[B4] Bandeira J. S. Antunes L. C. Soldatelli M. D. Sato J. R. Fregni F. Caumo W. (2019). Functional spectroscopy mapping of pain processing cortical areas during non-painful peripheral electrical stimulation of the accessory spinal nerve. Front. Hum. Neurosci. 13:200. 10.3389/fnhum.2019.00200 31263406 PMC6585570

[B5] Bantick S. J. Wise R. G. Ploghaus A. Clare S. Smith S. M. Tracey I. (2002). Imaging how attention modulates pain in humans using functional MRI. Brain 125, 310–319. 10.1093/brain/awf022 11844731

[B6] Becker M. (2021). MLR. Available online at: https://mlr3book.mlr-org.com/index.html (accessed November 26, 2021).

[B7] Benerradi J. A. Maior H. Marinescu A. Clos J. L. Wilson M. (2019). Exploring machine learning approaches for classifying mental workload using FNIRS data from HCI tasks, in Proceedings of the Halfway to the Future Symposium 2019 (New York, NY: Association for Computing Machinery). 10.1145/3363384.3363392

[B8] Birnie K. A. Chambers C. T. Spellman C. M. (2017). Mechanisms of distraction in acute pain perception and modulation. Pain 158, 1012–1013. 10.1097/j.pain.0000000000000913 28514252

[B9] Biswas S. A. Konar A. Basak P. (2017). Effect of disturbance in working memory performace: an fNIRs study, in 2017 Third International Conference on Biosignals, Images and Instrumentation (ICBSII) (Chennai, India), 1–6. 10.1109/ICBSII.2017.8082273

[B10] Burns C. G. Fairclough S. H. (2015). Use of auditory event-related potentials to measure immersion during a computer game. Int. J. Hum. Comput. Stud. 73, 107–114. 10.1016/j.ijhcs.2014.09.002

[B11] Causse M. Chua Z. Peysakhovich V. Del Campo N. Matton N. (2017). Mental workload and neural efficiency quantified in the prefrontal cortex using fNIRS. Sci. Rep. 7:5222. 10.1038/s41598-017-05378-x 28701789 PMC5507990

[B12] Chawla N. V. Bowyer K. W. Hall L. O. Kegelmeyer W. P. (2002). SMOTE: synthetic minority over-sampling technique. J. Artif. Int. Res. 16, 321–357. 10.1613/jair.95324088532

[B13] Chayadi E. McConnell B. L. (2019). Gaining insights on the influence of attention, anxiety, and anticipation on pain perception. J. Pain Res. 12, 851–864. 10.2147/JPR.S176889 30881096 PMC6402711

[B14] Chen L. Zhang A. Lou X. (2019). Cross-subject driver status detection from physiological signals based on hybrid feature selection and transfer learning. Expert Syst. Appl. 137, 266–280. 10.1016/j.eswa.2019.02.005

[B15] Combrisson E. Jerbi K. (2015). Exceeding chance level by chance: the caveat of theoretical chance levels in brain signal classification and statistical assessment of decoding accuracy. J. Neurosci. Methods 250, 126–136. 10.1016/j.jneumeth.2015.01.010 25596422

[B16] Cowley B. U. Palomäki J. Tammi T. Frantsi R. Inkil,ä V.-P. Lehtonen N.. (2019). Flow experiences during visuomotor skill acquisition reflect deviation from a power-law learning curve, but not overall level of skill. Front. Psychol. 10:1126. 10.3389/fpsyg.2019.01126 31156519 PMC6530424

[B17] Csikszentmihalyi M. (1990). Flow: The Psychology of Optimal Experience. New York, NY: Harper Perennial.

[B18] Cui X. Bray S. Reiss A. L. (2010). Functional near infrared spectroscopy (NIRS) signal improvement based on negative correlation between oxygenated and deoxygenated hemoglobin dynamics. Neuroimage 49, 3039–3046. 10.1016/j.neuroimage.2009.11.050 19945536 PMC2818571

[B19] Cui X. Bryant D. M. Reiss A. L. (2012). NIRS-based hyperscanning reveals increased interpersonal coherence in superior frontal cortex during cooperation. Neuroimage 59, 2430–2437. 10.1016/j.neuroimage.2011.09.003 21933717 PMC3254802

[B20] de Sampaio Barros M. F. Araújo-Moreira F. M. Trevelin L. C. Radel R. (2018). Flow experience and the mobilization of attentional resources. Cogn. Affect. Behav. Neurosci. 18, 81–823. 10.3758/s13415-018-0606-4 29736679

[B21] Dobbins C. Fairclough S. (2018). Signal processing of multimodal mobile lifelogging data towards detecting stress in real-world driving. IEEE Trans. Mob. Comput. 18, 632–644. 10.1109/TMC.2018.284015327295638

[B22] Ewing K. C. Fairclough S. H. Gilleade K. (2016). Evaluation of an adaptive game that uses EEG measures validated during the design process as inputs to a biocybernetic loop. Front. Hum. Neurosci. 10:223. 10.3389/fnhum.2016.00223 27242486 PMC4870503

[B23] Fairclough S. H. Burns C. Kreplin U. (2018). FNIRS activity in the prefrontal cortex and motivational intensity: impact of working memory load, financial reward, and correlation-based signal improvement. Neurophotonics 5, 1–10. 10.1117/1.NPh.5.3.035001 30035151 PMC6041856

[B24] Fairclough S. H. Stamp K. Dobbins C. Poole H. M. (2020). Computer games as distraction from pain: effects of hardware and difficulty on pain tolerance and subjective immersion. Int. J. Hum. Comput. Stud. 139:102427. 10.1016/j.ijhcs.2020.102427

[B25] Fernandez Rojas R. Huang X. Ou K. L. (2019). A machine learning approach for the identification of a biomarker of human pain using fNIRS. Sci. Rep. 9:5645. 10.1038/s41598-019-42098-w 30948760 PMC6449551

[B26] Fernández A. Garcia S. Herrera F. Chawla N. V. (2018). SMOTE for learning from imbalanced data: progress and challenges, marking the 15-year anniversary. J. Artif. Int. Res. 61, 863–905. 10.1613/jair.1.11192

[B27] Fernández H. D. Koji M. Kondo K. (2017). Adaptable game experience based on player's performance and EEG, in Proceeding 2017 Nicograph International, (Kyoto, Japan), 1–8. 10.1109/NICOInt.2017.11

[B28] Forte G. Favieri F. Casagrande M. (2019). Heart rate variability and cognitive function: a systematic review. Front. Neurosci. 13:710. 10.3389/fnins.2019.00710 31354419 PMC6637318

[B29] Gateau T. Durantin G. Lancelot F. Scannella S. Dehais F. (2015). Real-time state estimation in a flight simulator using fNIRS. PLoS ONE 10:e0121279. 10.1371/journal.pone.0121279 25816347 PMC4376943

[B30] Gentile E. Brunetti A. Ricci K. Delussi M. Bevilacqua V. de Tommaso M. (2020). Mutual interaction between motor cortex activation and pain in fibromyalgia: EEG-fNIRS study. PLoS ONE 15:e0228158. 10.1371/journal.pone.0228158 31971993 PMC6977766

[B31] Harmat L. de Manzano Ö. Theorell T. Högman L. Fischer H. Ullén F. (2015). Physiological correlates of the flow experience during computer game playing. Int. J. Psychophysiol. 97, 1–7. 10.1016/j.ijpsycho.2015.05.001 25956190

[B32] Hart S. G. Staveland L. E. (1988). Development of the NASA-TLX (task load index): results of empirical and theoretical research, in Human Mental Workload, eds Hancock P. A. Meshkati N. (Amsterdam: North-Holland), 139–183. 10.1016/S0166-4115(08)62386-9

[B33] Hong K.-S. Khan M. J. Hong M. J. (2018). Feature extraction and classification methods for hybrid fNIRS-EEG brain-computer interfaces. Front. Hum. Neurosci. 12:246. 10.3389/fnhum.2018.00246 30002623 PMC6032997

[B34] Inan G. Inal S. (2019). The impact of 3 different distraction techniques on the pain and anxiety levels of children during venipuncture: a clinical trial. Clin. J. Pain 35, 140–147. 10.1097/AJP.0000000000000666 30362982

[B35] Islam M. J. Wu Q. M. J. Ahmadi M. Sid-Ahmed M. A. (2007). Investigating the performance of naive- bayes classifiers and K- nearest neighbor classifiers, in 2007 International Conference on Convergence Information Technology, (Gwangju, South Korea), 1541–1546. 10.1109/ICCIT.2007.148

[B36] Jameson E. Trevena J. Swain N. (2011). Electronic gaming as pain distraction. Pain Res. Manag. 16, 27–32. 10.1155/2011/856014 21369538 PMC3052404

[B37] Jasper H. H. (1958). Report of the committee on methods of clinical examination in electroencephalography. Electroencephalogr. Clin. Neurophysiol. 10, 370–375. 10.1016/0013-4694(58)90053-1

[B38] Jennett C. Cox A. L. Cairns P. Dhoparee S. Epps A. Tijs T.. (2008). Measuring and defining the experience of immersion in games. Int. J. Hum. Comput. Stud. 66, 641–661. 10.1016/j.ijhcs.2008.04.004

[B39] Kamran M. A. Mannan M. M. Jeong M. Y. (2016). Cortical signal analysis and advances in functional near-infrared spectroscopy signal: a review. Front. Hum. Neurosci. 10:261. 10.3389/fnhum.2016.00261 27375458 PMC4899446

[B40] Keller J. Landhäußer A. (2012). The flow model revisited, in Advances in Flow Research eds Engeser S. (New York, NY: Springee), 51–64. 10.1007/978-1-4614-2359-1_3

[B41] Koenig J. Jarczok M. N. Ellis R. J. Hillecke T. K. Thayer J. F. (2014). Heart rate variability and experimentally induced pain in healthy adults: a systematic review. Eur. J. Pain 18, 301–314. 10.1002/j.1532-2149.2013.00379.x 23922336

[B42] Koller D. Goldman R. D. (2012). Distraction techniques for children undergoing procedures: a critical review of pediatric research. J. Pediatr. Nurs. 27, 652–681. 10.1016/j.pedn.2011.08.001 21925588

[B43] Kononenko I. Šimec E. Robnik-Šikonja M. (1997). Overcoming the myopia of inductive learning algorithms with RELIEFF. Appl. Intell. 7, 39–55. 10.1023/A:1008280620621

[B44] Kucyi A. Salomons T. V. Davis K. D. (2013). Mind wandering away from pain dynamically engages antinociceptive and default mode brain networks. Proc. Natl. Acad. Sci. U.S.A. 110, 18692 LP−18697. 10.1073/pnas.1312902110 24167282 PMC3832014

[B45] Kwon J. Shin J. Im C.-H. (2020). Toward a compact hybrid brain-computer interface (BCI): performance evaluation of multi-class hybrid EEG-fNIRS BCIs with limited number of channels. PLoS ONE 15:e0230491. 10.1371/journal.pone.0230491 32187208 PMC7080269

[B46] Lang M. Binder M. Richter J. Schratz P. Pfisterer F. Coors S.. (2019). mlr3: a modern object-oriented machine learning framework in R. J. Open Source Softw. 4:1903. 10.21105/joss.01903

[B47] Law E. F. Dahlquist L. M. Sil S. Weiss K. E. Herbert L. J. Wohlheiter K.. (2011). Videogame distraction using virtual reality technology for children experiencing cold pressor pain: the role of cognitive processing. J. Pediatr. Psychol. 36, 84–94. 10.1093/jpepsy/jsq063 20656761 PMC3107585

[B48] Legrain V. Damme S. V. Eccleston C. Davis K. D. Seminowicz D. A. Crombez G. (2009). A neurocognitive model of attention to pain: behavioral and neuroimaging evidence. Pain 144, 230–232. 10.1016/j.pain.2009.03.020 19376654

[B49] Legrain V. Mancini F. Sambo C. F. Torta D. M. Ronga I. Valentini E. (2012). Cognitive aspects of nociception and pain. Bridging neurophysiology with cognitive psychology. Clin. Neurophysiol. 42, 325–336. 10.1016/j.neucli.2012.06.003 23040703

[B50] Liu C. Agrawal P. Sarkar N. Chen S. (2009). Dynamic difficulty adjustment in computer games through real-time anxiety-based affective feedback. Int. J. Hum. Comp. Interact. 25, 506–529. 10.1080/10447310902963944

[B51] Lopez-Martinez D. Peng K. Lee A. Borsook D. Picard R. (2019). Pain detection with fNIRS-measured brain signals: a personalized machine learning approach using the wavelet transform and bayesian hierarchical modeling with dirichlet process priors, in International Conference on Affective Computing and Intelligent Interaction (ACII) Workshop on Recognition, Treatment and Management of Pain and Distress. (Cambridge, UK), 10.1109/ACIIW.2019.8925076

[B52] Lu J. Yan H. Chang C. Wang N. (2020). Comparison of machine learning and deep learning approaches for decoding brain computer interface: an fNIRS study, in Intelligent Information Processing X, eds Shi Z. Vadera S. Chang E. (Cham: Springer International Publishing), 192–201. 10.1007/978-3-030-46931-3_18

[B53] Maimon O. Rokach L. (2010). Data Mining and Knowledge Discovery Handbook, 2nd edn. New York, NY: Springer. 10.1007/978-0-387-09823-4

[B54] Malloy K. M. Milling L. S. (2010). The effectiveness of virtual reality distraction for pain reduction: a systematic review. Clin. Psychol. Rev. 30, 1011–1018. 10.1016/j.cpr.2010.07.001 20691523

[B55] Martey R. M. Kenski K. Folkestad J. Feldman L. Gordis E. Shaw A.. (2014). Measuring game engagement: multiple methods and construct complexity. Simul. Gaming 45, 528–547. 10.1177/1046878114553575

[B56] Matthews G. (2016). Multidimensional profiling of task stress states for human factors: a brief review. Hum. Fact. 58, 801–813. 10.1177/0018720816653688 27329044

[B57] Meidenbauer K. L. Choe K. W. Cardenas-Iniguez C. Huppert T. J. Berman M. G. (2021). Load-dependent relationships between frontal fNIRS activity and performance: a data-driven PLS approach. Neuroimage 230:117795. 10.1016/j.neuroimage.2021.117795 33503483 PMC8145788

[B58] Metz A. J. Wolf M. Achermann P. Scholkmann F. (2015). A new approach for automatic removal of movement artifacts in near-infrared spectroscopy time series by means of acceleration data. Algorithms 8, 1052–1075. 10.3390/a8041052

[B59] Michailidis L. Balaguer-Ballester E. He X. (2018). Flow and immersion in video games: the aftermath of a conceptual challenge. Front. Psychol. 9:1682. 10.3389/fpsyg.2018.01682 30233477 PMC6134042

[B60] Morris L. D. Louw Q. A. Grimmer-Somers K. (2009). The effectiveness of virtual reality on reducing pain and anxiety in burn injury patients: a systematic review. Clin. J. Pain 25, 815–826. 10.1097/AJP.0b013e3181aaa909 19851164

[B61] Naseer N. Hong K. S. (2015). fNIRS-based brain-computer interfaces: a review. Front. Hum. Neurosci. 9:3. 10.3389/fnhum.2015.00003 25674060 PMC4309034

[B62] Naseer N. Qureshi N. K. Noori F. M. Hong K.-S. (2016). Analysis of different classification techniques for two-class functional near-infrared spectroscopy-based brain-computer interface. Intell. Neurosci. 2016:5480760. 10.1155/2016/5480760 27725827 PMC5048089

[B63] Nilsson S. Enskär K. Hallqvist C. Kokinsky E. (2013). Active and passive distraction in children undergoing wound dressings. J. Pediatr. Nurs. 28, 158–166. 10.1016/j.pedn.2012.06.003 22819747

[B64] Pinti P. Scholkmann F. Hamilton A. Burgess P. Tachtsidis I. (2019). Current status and issues regarding pre-processing of fNIRS neuroimaging data: an investigation of diverse signal filtering methods within a general linear model framework. Front. Hum. Neurosci. 12:505. 10.3389/fnhum.2018.00505 30687038 PMC6336925

[B65] Poole H. M. (2014). unpublished poster presentation. World Congress of Pain Buenos Aires.

[B66] Raudenbush B. Koon J. Cessna T. McCombs K. (2009). Effects of playing video games on pain response during a cold pressor task. Percept. Mot. Skills 108, 439–448. 10.2466/pms.108.2.439-448 19544949

[B67] Richter M. Gendolla G. H. E. Wright R. A. (2016). Three decades of research on motivational intensity theory: what we have learned about effort and what we still don't know. Adv. Motiv. Sci. 3, 149–186. 10.1016/bs.adms.2016.02.001

[B68] Sarkar A. Cooper S. (2019). Transforming game difficulty curves using function composition, in CHI '19: Proceedings of the 2019 CHI Conference on Human Factors in Computing Systems, (Glasgow, UK), 1–7. 10.1145/3290605.3300781

[B69] Torta D. M. Legrain V. Mouraux A. Valentini E. (2017). Attention to pain! A neurocognitive perspective on attentional modulation of pain in neuroimaging studies. Cortex 89, 120–134. 10.1016/j.cortex.2017.01.010 28284849 PMC7617013

[B70] Trost Z. France C. Anam M. Shum C. (2021). Virtual reality approaches to pain: toward a state of the science. Pain 162, 325–331. 10.1097/j.pain.0000000000002060 32868750

[B71] Urbanowicz R. J. Meeker M. La Cava W. Olson R. S. Moore J. H. (2018). Relief-based feature selection: introduction and review. J. Biomed. Inform. 85, 189–203. 10.1016/j.jbi.2018.07.014 30031057 PMC6299836

[B72] Verdière K. J. Roy R. N. Dehais F. (2018). Detecting pilot's engagement using fnirs connectivity features in an automated vs. manual landing scenario. Front. Hum. Neurosci. 12:6. 10.3389/fnhum.2018.00006 29422841 PMC5788966

[B73] Von Baeyer C. L. Piira T. Chambers C. T. Trapanotto M. Zeltzer L. K. (2005). Guidelines for the cold pressor task as an experimental pain stimulus for use with children. J. Pain 6, 218–227. 10.1016/j.jpain.2005.01.349 15820909

[B74] Williams A. Ishimine P. (2016). Non-pharmacologic management of pain and anxiety in the pediatric patient. Curr. Emerg. Hosp. Med. Rep. 4, 26–31. 10.1007/s40138-016-0090-5

[B75] Wohlheiter K. A. Dahlquist L. M. (2013). Interactive versus passive distraction for acute pain management in young children: the role of selective attention and development. J. Pediatr. Psychol. 38, 202–212. 10.1093/jpepsy/jss108 23092971

[B76] Wosiak A. Zakrzewska D. (2018). Integrating correlation-based feature selection and clustering for improved cardiovascular disease diagnosis. Complexity 2018, 2520706. 10.1155/2018/2520706

[B77] Xue S. Wu M. Kolen J. Aghdaie N. Zaman K. A. (2017). Dynamic difficulty adjustment for maximized engagement in digital games, in Proceedings of the 26th International Conference on World Wide Web Companion WWW '17 Companion (Geneva: International World Wide Web Conferences Steering Committee), 465–471. 10.1145/3041021.3054170

[B78] Yannakakis G. Spronck P. Loiacono D. Andre E. (2013). Player modeling, in Artificial and Computational Intelligence in Games, eds Lucas S. M. Mateas M. Preuss M. Spronck P. Togelius J. (Saarsbrucken: Dagstuhl Publishing), 45–59.

[B79] Zohaib M. (2018). Dynamic difficulty adjustment (DDA) in computer games: a review. Adv. Hum. Comp. Interact. 2018:5681652. 10.1155/2018/5681652

