# Supplementary material for: Classification of Game Demand and the Presence of Experimental Pain Using Functional Near-Infrared Spectroscopy
Source: Front Neuroergon. 2021 Dec 21;2:695309. doi: 10.3389/fnrgo.2021.695309 (PMC10790923; doi:10.3389/fnrgo.2021.695309)
Supplement: Supplementary file 1 [file Data_Sheet_1.pdf]

Appendix 1 – Optimal tuning parameters for the classification of Game Demand (Easy vs Hard) using fNIRS features for the kNN, RF and SVM algorithms, across all ten folds. CE = classification error, MTRY = number of variables to split at each node

Algorithm	k	Distance	CE
kNN	11	1	0.00417
	3	1	0.00833
	3	1	0.00833
	20	1	0.00417
	20	1	0
	20	1	0.00417
	20	1	0
	20	1	0.07917
	20	1	0
	20	1	0

Algorithm	MTRY	Number of Threads	Number of Trees	CE
RF	1	1	10	0
	2	1	20	0
	2	20	10	0.01250
	2	10	20	0
	2	10	20	0
	2	20	10	0
	1	20	20	0
	2	1	10	0.07083
	2	20	10	0
	1	1	10	0

Algorithm	Cost	Gamma	Kernel	Degree	CE
SVM	100000	0.00001	radial	NA	0.22500
	50000	0.00001	polynomial	1	0.32917
	100000	0.00001	radial	NA	0.15000
	100000	0.00001	radial	NA	0.22917
	0.00001	100000	polynomial	1	0.17500
	100000	0.00001	radial	NA	0.18750
	100000	0.00001	polynomial	1	0.23333
	0.00001	100000	polynomial	1	0.27500
	0.00001	100000	polynomial	1	0.20833

	100000	0.00001	radial	NA	0.20

Appendix 2 – Optimal tuning parameters for the classification of Game Demand (Easy vs Hard) using HR features for the kNN, RF and SVM algorithms, across all ten folds. CE = classification error, MTRY = number of variables to split at each node

**Algorithm**	**k**	**Distance**	**CE**
**kNN**	20	1	0.39412
	20	1	0.35294
	11	3	0.39431
	3	3	0.41672
	20	1	0.40062
	20	3	0.45615
	11	3	0.42115
	11	5	0.39459
	3	1	0.37765
	11	1	0.40946

**Algorithm**	**MTRY**	**Number of Threads**	**Number of Trees**	**CE**
**RF**	2	20	20	0.38529
	1	20	10	0.39412
	2	20	20	0.38677
	2	1	10	0.41975
	2	10	10	0.39039
	2	1	10	0.44909
	2	10	20	0.47276
	1	20	10	0.38675
	1	1	10	0.38076
	2	20	20	0.38535

**Algorithm**	**Cost**	**Gamma**	**Kernel**	**Degree**	**CE**
**SVM**	50000	50000	radial	NA	0.38529
	100000	100000	radial	NA	0.39118
	0.00001	100000	polynomial	4	0.41015
	100000	100000	polynomial	1	0.43983
	50000	50000	radial	NA	0.36921
	100000	100000	polynomial	1	0.42782
	100000	50000	polynomial	1	0.45276
	0.00001	100000	radial	NA	0.35641
	0.00001	100000	radial	NA	0.41218

	50000	0.00001	polynomial	1	0.39637

Appendix 3 – Optimal tuning parameters for the classification of Easy Game Demand (Pain vs No Pain) using fNIRS features for the kNN, RF and SVM algorithms, across all ten folds. CE = classification error, MTRY = number of variables to split at each node

Algorithm	k	Distance	CE
kNN	3	1	0.04444
	3	1	0.05556
	3	1	0.01111
	3	1	0.02222
	3	1	0.10000
	11	1	0.02222
	3	1	0.05556
	3	1	0.00000
	3	1	0.02222
	3	1	0.05556

Algorithm	MTRY	Number of Threads	Number of Trees	CE
RF	1	10	20	0.03333
	2	20	20	0.07778
	1	1	20	0.01111
	2	10	10	0.03333
	2	20	20	0.20
	2	1	20	0
	2	20	20	0.04444
	2	1	20	0.04444
	2	20	20	0.05556
	2	20	10	0.03333

Algorithm	Cost	Gamma	Kernel	Degree	CE
SVM	100000	0.00001	radial	NA	0.26667
	100000	100000	polynomial	2	0.18889
	50000	0.00001	polynomial	1	0.23333
	100000	0.00001	radial	NA	0.31111
	100000	50000	polynomial	2	0.24444
	100000	0.00001	polynomial	4	0.41111
	100000	0.00001	radial	NA	0.30
	100000	0.00001	radial	NA	0.26667
	50000	0.00001	polynomial	1	0.22222

	100000	50000	polynomial	2	0.15556

Appendix 4 – Optimal tuning parameters for the classification of Hard Game Demand (Pain vs No Pain) using fNIRS features for the kNN, RF and SVM algorithms, across all ten folds. CE = classification error, MTRY = number of variables to split at each node

Algorithm	k	Distance	CE
kNN	3	1	0.10
	3	1	0.07778
	3	1	0.00
	3	1	0.18889
	3	1	0.12222
	11	1	0.06667
	3	1	0.02222
	3	1	0.14444
	3	1	0.10
	11	1	0.05556

Algorithm	MTRY	Number of Threads	Number of Trees	CE
RF	2	10	20	0.03333
	1	1	10	0.07778
	1	1	10	0.04444
	1	10	10	0.17778
	1	1	20	0.18889
	2	20	20	0.10
	2	20	20	0.04444
	2	10	10	0.11111
	2	1	10	0.07778
	2	20	10	0.04444

Algorithm	Cost	Gamma	Kernel	Degree	CE
SVM	100000	100000	polynomial	2	0.25556
	50000	50000	polynomial	2	0.16667
	0.00001	50000	polynomial	2	0.18889
	0.00001	100000	polynomial	2	0.25556
	50000	100000	polynomial	1	0.21111
	100000	100000	polynomial	2	0.18889
	0.00001	50000	polynomial	4	0.10
	50000	100000	polynomial	4	0.20
	50000	0.00001	polynomial	1	0.15556

	50000	50000	polynomial	2	0.18889

Appendix 5 – Optimal tuning parameters for the classification of Easy Game Demand (Pain vs No Pain) using HR features for the kNN, RF and SVM algorithms, across all ten folds. CE = classification error, MTRY = number of variables to split at each node

Algorithm	k	Distance	CE
kNN	3	3	0.41935
	11	1	0.41011
	11	3	0.47231
	20	5	0.46400
	3	3	0.44167
	3	3	0.39641
	20	1	0.45092
	20	5	0.34620
	3	3	0.47804
	11	1	0.39843

Algorithm	MTRY	Number of Threads	Number of Trees	CE
RF	2	20	1	0.43871
	2	10	1	0.41382
	1	10	1	0.45262
	1	20	1	0.44717
	1	20	10	0.42083
	1	20	1	0.46013
	2	10	10	0.45831
	1	1	20	0.33567
	1	1	20	0.39868
	1	10	20	0.40969

Algorithm	Cost	Gamma	Kernel	Degree	CE
SVM	50000	0.00001	polynomial	2	0.43548
	100000	0.00001	radial	NA	0.39387
	50000	0.00001	polynomial	2	0.41431
	50000	50000	radial	NA	0.43883
	0.00001	50000	radial	NA	0.41667
	50000	0.00001	polynomial	2	0.43301
	50000	100000	polynomial	1	0.44385
	100000	0.00001	radial	NA	0.33392
	0.00001	0.00001	polynomial	2	0.43426

	0.00001	100000	polynomial	2	0.39473

Appendix 6 – Optimal tuning parameters for the classification of Hard Game Demand (Pain vs No Pain) using HR features for the kNN, RF and SVM algorithms, across all ten folds. CE = classification error, MTRY = number of variables to split at each node

**Algorithm**	**k**	**Distance**	**CE**
**kNN**	20	3	0.42792
	20	1	0.42504
	11	3	0.42718
	11	1	0.45391
	3	1	0.38518
	11	1	0.45575
	3	3	0.45233
	20	5	0.41914
	11	1	0.47698
	3	3	0.41957

**Algorithm**	**MTRY**	**Number of Threads**	**Number of Trees**	**CE**
**RF**	1	20	10	0.41552
	2	10	10	0.40244
	2	10	1	0.43316
	1	10	1	0.43644
	2	1	10	0.38202
	1	20	1	0.42067
	1	1	20	0.41111
	1	10	1	0.40194
	1	1	20	0.46138
	1	1	10	0.42355

**Algorithm**	**Cost**	**Gamma**	**Kernel**	**Degree**	**CE**
**SVM**	50000	100000	polynomial	1	0.41573
	100000	50000	polynomial	2	0.45185
	100000	100000	polynomial	2	0.43039
	100000	0.00001	polynomial	4	0.44770
	100000	0.00001	polynomial	2	0.43439
	0.00001	0.00001	polynomial	2	0.45222
	50000	100000	polynomial	4	0.44640
	100000	100000	polynomial	2	0.43871
	0.00001	50000	polynomial	2	0.43373

	0.00001	50000	polynomial	1	0.41576
